# Assembly and co-occurrence networks of nitrogen-fixing bacteria associated with epiphyllous liverworts in fragmented tropical forests

**DOI:** 10.1093/ismeco/ycaf173

**Published:** 2025-09-27

**Authors:** Adriel M Sierra, Dennis Alejandro Escolástico-Ortiz, Charles E Zartman, Nicolas Derome, Connie Lovejoy, Juan Carlos Villarreal A

**Affiliations:** Département de Biologie, Université Laval, G1V 0A6, Québec, QC G1V 0A6, Canada; Institut de Biologie Intégrative et dês Systèmes (IBIS), Université Laval, Québec, QC G1V 0A6, Canada; Departamento de Biodiversidade, Instituto Nacional de Pesquisas da Amazônia, Av. André Araújo, 2936, Aleixo, Manaus, AM CEP 69060-001, Brazil; Département de Biologie, Université Laval, G1V 0A6, Québec, QC G1V 0A6, Canada; Institut de Biologie Intégrative et dês Systèmes (IBIS), Université Laval, Québec, QC G1V 0A6, Canada; Departamento de Biodiversidade, Instituto Nacional de Pesquisas da Amazônia, Av. André Araújo, 2936, Aleixo, Manaus, AM CEP 69060-001, Brazil; Département de Biologie, Université Laval, G1V 0A6, Québec, QC G1V 0A6, Canada; Institut de Biologie Intégrative et dês Systèmes (IBIS), Université Laval, Québec, QC G1V 0A6, Canada; Département de Biologie, Université Laval, G1V 0A6, Québec, QC G1V 0A6, Canada; Institut de Biologie Intégrative et dês Systèmes (IBIS), Université Laval, Québec, QC G1V 0A6, Canada; Département de Biologie, Université Laval, G1V 0A6, Québec, QC G1V 0A6, Canada; Institut de Biologie Intégrative et dês Systèmes (IBIS), Université Laval, Québec, QC G1V 0A6, Canada

**Keywords:** Amazon forest, co-occurrence network, diazotrophic community, habitat loss, key species, metacommunity, nitrogen-fixation, phyllosphere

## Abstract

Understanding the spatial dynamics of plant-associated microbial communities is increasingly urgent in the context of habitat loss and the biodiversity crisis. However, the influence of reduced habitat size and connectivity on the assembly mechanisms underlying microbial associations is fundamental to advancing microbial ecology and conservation. In the Brazilian Amazon, we investigated nitrogen-fixing (diazotrophic) bacterial communities associated with two epiphyllous liverworts, *Cololejeunea surinamensis* and *Radula flaccida*, across 11 forest sites within the Biological Dynamics of Forest Fragments Project landscape. Using amplicon sequencing targeting the nitrogenase gene (*nif*H), we characterized diazotroph community diversity, inferred assembly mechanisms through null models, and analyzed co-occurrence network structure. Host-specific associations were evident: *C. surinamensis* predominantly hosted *Hassallia*, while *R. flaccida* was primarily associated with *Fischerella*. Despite habitat fragmentation, diazotrophic richness and composition remained similar across habitats of different sizes, consistent with strong homogenizing dispersal. Network analyses revealed that smaller fragments harbored more modular communities with fewer module hubs, pronounced shifts in key species relative abundance, and reduced network robustness. Our findings underscore the influence of habitat size on the stability of liverwort-associated diazotrophs, with smaller fragments exhibiting lower key species specificity and disruption of microbe-microbe interactions. Our results emphasize the importance of conserving large, connected forest habitats to maintain the functional integrity of phyllosphere N-fixing microbiota.

## Graphical Abstract

**Graphical Abstract:**
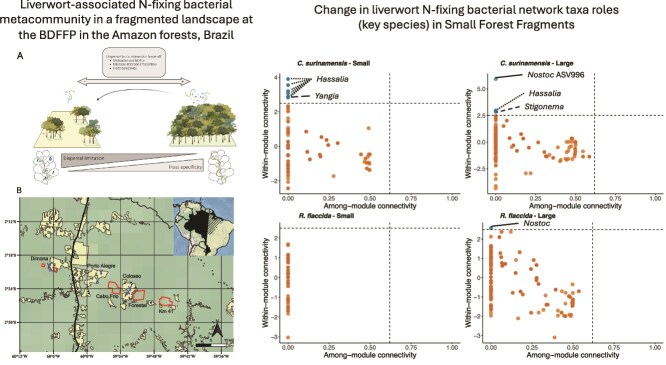

## Introduction

Tropical forests are among the most biodiverse ecosystems on Earth where intricate biotic interactions take place, sustaining biodiversity and providing ecosystem functions such as productivity and nutrient cycling [1–4]. Among these interactions, plant-associated nitrogen-fixing microbes (diazotrophs) are critical contributors to the nitrogen cycle (N-cycle) [5, 6]. Diazotrophs associate with all bryophyte lineages, and by fixing nitrogen, they contribute substantially to the local ecosystem N budget [7–9]. In tropical forests, epiphyllous (leaf-inhabiting) bryophytes host diverse microbial communities, including associated diazotrophs, forming complex and multifunctional holobionts [10–13].

Above-ground biological nitrogen fixation (BNF) by epiphyllous-associated diazotrophs contributes 30%–40% of the nitrogen budget in lowland tropical forests [5, 6]. BNF rates vary widely in response to abiotic factors like climate and resource availability, and biotic factors including host identity and holobiont chemical signaling [5, 6, 8, 11, 13, 14]. Host-derived exudates, such as hormogonium-inducing factors, can further modulate diazotrophs’ associations and activity under variable environmental conditions [8]. Broad environmental changes associated with land conversion from forests to pastures, shifted soil diazotroph community composition, potentially impacting BNF activity [15, 16]. In fragmented forests, both deterministic and stochastic processes may drive hosts to associate with locally available, less specific, and less efficient diazotrophs [17–20]. In this context, characterizing the assembly and stability of nitrogen-fixing microbial communities is essential for understanding how land-use change impacts symbiotic relationships, as altered dynamics may compromise BNF [8, 21, 22].

Deforestation is the principal cause of the fragmentation of 70% of remaining forest habitats worldwide, increasing species’ proximity to forest edges [23]. Habitat fragmentation, a by-product of deforestation, disrupts species dispersal, and degrades local environmental conditions, resulting in the gradual loss of species and biotic interactions, like mutualisms [24, 25]. While the negative impacts of forest fragmentation on species richness and community composition are well-documented [26, 27], its broader consequences for the stability of host–microbe interactions remain poorly understood [24, 25, 28]. Moreover, distribution patterns of plants with N-fixing microbial partners suggest that dispersal limitation of the associated microbes may further constrain species ranges compared to plants that do not rely on this microbial association [29].

The mechanisms underlying the assembly and partner specificity of plant microbiota in fragmented landscapes can be investigated through the lens of metacommunity and network theory. The metacommunity framework elucidates the processes structuring host–microbe communities along the axes of dispersal and environmental heterogeneity, treating hosts as dynamic patches where local microbial communities are shaped by the regional pools through dispersal [20, 30–33]. At the same time, deterministic feedback from the host and environmental conditions further influence microbial assembly and specificity [20, 32, 34].

Network theory complements the metacommunity framework by further characterizing microbial associations as the degree of taxa co-occurrences in a community [35, 36]. Microbial co-occurrence networks enable the inference of microbial association patterns and identify their specific taxa roles [37]. Taxa highly connected in a microbial network (key species) promote stability by supporting other taxa’s fitness through positive feedback [38]. Moreover, network properties, such as modularity and cohesion, influence the extent to which communities respond to disturbance and their capacity for recovery [35]. These properties reflect aspects of community stability and robustness. Thus, network theory provides a comprehensive framework to assess the stability and specialization of host-microbe under the pressures of habitat loss [35, 39, 40].

**Figure 1:**
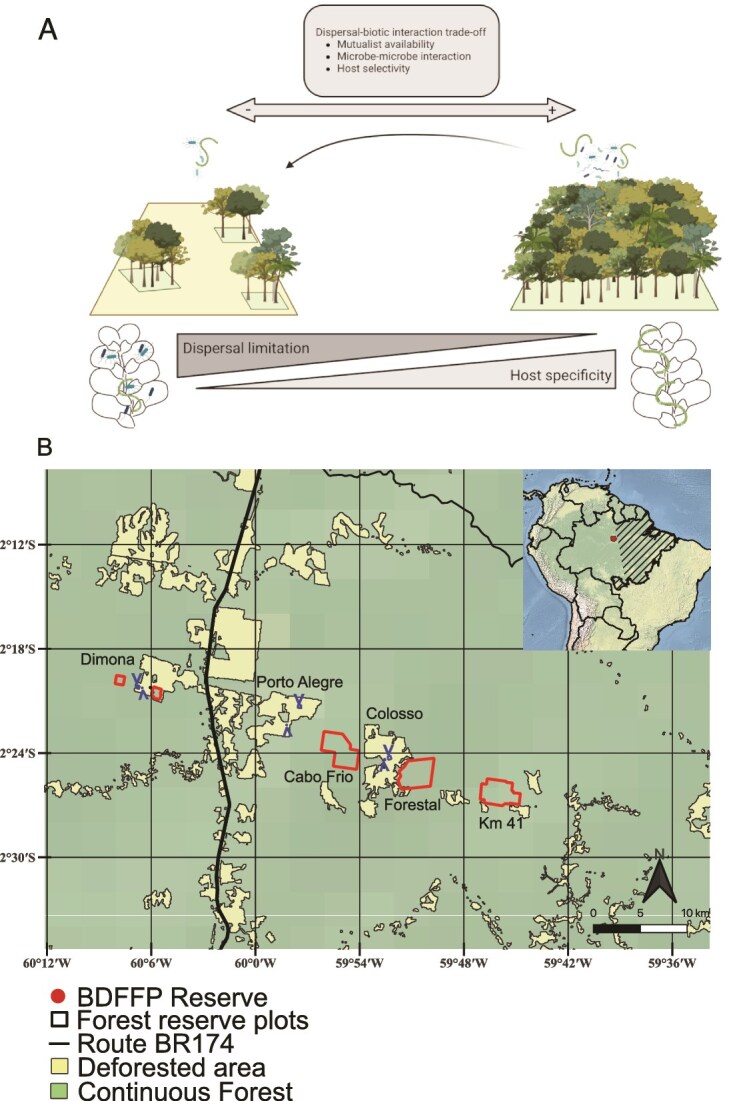
Study conceptual framework and site map. (A) Hypothetical shifts in liverwort-microbiota associations across an Amazonian fragmented landscape. In smaller, isolated fragments, limited microbial availability and dispersal constraints (represented with an arrow) are expected to weaken microbe–microbe interactions and reduce host selectivity. In contrast, continuous forests may facilitate more specific and stable host–microbe associations due to a more diverse and connected microbial pool, and stronger biotic filtering. “Created in BioRender. Villarreal, J. (2024) https://BioRender.com/n61i393” (B) Map of the study area within the BDFFP, Amazonas, Brazil. Forest fragments of 1-ha and 10-ha (within or adjacent to deforested areas) and 100-ha are distributed across three reserves (Dimona, Porto Alegre, and Colosso) and are separated by deforested matrix from continuous mature forests located in four additional reserves.

In this study, we evaluated the composition and co-occurrence network of diazotrophs associated with epiphyllous liverworts across the experimentally fragmented landscape of the Biological Dynamic Forest Fragment Project (BDFFP). Leveraging the long-term experimental forest sites of the BDFFP in the Brazilian Amazonian forests, we examined how habitat size shapes host-microbe community structure in small (1- and 10-ha) and large (100-ha) forest fragments and continuous forest (Fig. 1A). We assessed liverwort-associated diazotrophs using amplicon sequencing targeting the nitrogenase reductase gene (*nif*H) complex, to address two questions: (i) Which mechanisms determine host-diazotroph composition in a fragmented landscape? (ii) How do habitat size influence diazotrophic community network stability, based on the co-occurrence patterns and robustness?

We hypothesized that diazotrophs’ assembly is constrained by dispersal limitations and reduced host-microbe specificity in smaller fragments. Using quantitative community matrices and network theory, we evaluate the stability and robustness of diazotroph associations by comparing network properties and simulating species loss scenarios [41, 42]. We predicted that liverwort diazotrophic networks would become more compartmentalized (higher modularity), and that network robustness would decline disproportionately following the removal of key species compared to random species loss.

## Materials and methods

### Study site

The BDFFP is located in central Brazilian Amazonia (2°30 S, 60°02 W), ~80 km north of Manaus, along the BR-174 highway. The BDFFP consists of *terra firme* (non-flooded) lowland rainforest on nutrient-poor soils, with elevations ranging between 50 and 150 meters above sea level [43]. The region experiences rainfall throughout the year, with an annual precipitation of 1900 to 2550 mm [44]. In the BDFFP, forest fragments were experimentally delimited and isolated in 1980 among three adjacent cattle ranch reserves (Dimona, Porto Alegre, and Colosso in the Esteio farm). Forest fragments comprise multiple replicates of 1-, 10-, and 100-ha, separated by 70 to 1000 m from continuous forests across the landscape (Fig. 1B). The matrix in the landscape was composed of cattle pasture from 1980 to 1995, but secondary forest dominated by *Vismia* spp. and *Cecropia* spp. has replaced the pasture since 2015 [43].

We used the epiphyllous liverworts as a system for studying plant-microbe interactions, as their restriction to the leaf surface provides a well-defined microhabitat that facilitates standardized sampling across sites [45]. We sampled a total of 11 forest sites in May of 2017, including six small patches: 1-ha (*n* = 3) and 10-ha (*n* = 3); and five large patches: 100-ha (*n* = 1) and continuous forests (*n* = 4) (Fig. 1B). We selected two common epiphyllous liverwort species inhabiting the leaves of young trees, shrubs, and understory palms, *Radula flaccida* Lindenb. & Gott., (Radulaceae) and *Cololejeunea surinamensis* Tixier (Lejeuneaceae). Specifically, we gathered leaf samples from four dominant understory plants (predominately the shrub species *Duguetia flagellaris* Huber, and *Rinorea racemosa* (Mart.) Kuntze, and the palms *Astrocaryum sciophilum* (Miq.) Pulle, and *Attalea attaleoides* (Barb. Rodr.) Wess. Boer), harboring the targeted liverworts. Samples were found between one to two meters height above ground, and spatially spaced at least 10 m apart from one another, within the 1-ha long-term study plots at each BDFFP forest site. In the laboratory, using a dissecting scope, we carefully separated 0.5–1 g of the bryophyte tissue from the leaf surface, avoiding contamination from host plant tissue or other epiphyllous species, and stored it in sterile silica gel, which prevents DNA degradation. In total, 89 and 87 samples were collected for *C. surinamensis* and *R. flaccida*, respectively, resulting in 176 microbial communities equally distributed in small (1- and 10-ha; *C. surinamensis n* = 36 and *R. flaccida n* = 37) and large (100-ha and continuous; *C. surinamensis n* = 34 and *R. flaccida n* = 39) forest habitats (Table S1).

### DNA extraction and amplicon sequencing

Bryophyte tissue was flash-frozen in liquid nitrogen to facilitate pulverization prior to total genomic DNA extraction using an E.Z.N.A. Plant DNA DS Mini kit (Omega Bio-tek, Inc., Norcross, GA, USA). Amplicon sequencing libraries were prepared with a two-step dual-indexed PCR approach for Illumina sequencing with specific primers fused to Illumina TruSeq sequencing adapters. We amplified the nitrogenase enzyme coding region *nif*H with the primer set Ueda19F and R6, due to its higher specificity to the nitrogenase reductase *nif*H gene reduces unspecific amplification bias [46, 47]. Dual indices and Illumina flow cell adaptors to all amplicons were subsequently added. PCR reactions followed the TopTaq DNA Polymerase protocol (Qiagen, Venlo, The Netherlands) previously described [9], with the following PCR program cycle: 3 min 94°C, followed by 35 cycles of 30 s 94°C, 30 s 52°C, 60 s 72°C, and 10 min 72°C. Sequencing was performed with a 2 × 300 bp run using an Illumina MiSeq sequencer (Illumina, San Diego, CA, USA), at the platform of genomic analysis of the Institute of Integrative Biology and Systems, Université Laval, Québec, Canada (2 × 300 bp run).

### Bioinformatics

Amplicon sequence processing and statistical analyses were conducted using the R software (R Core Team v4.3.2), via the RStudio interface [48]. We evaluated the number of reads generated for the samples and removed the primer sequences and the Illumina adaptors using the software *cutadapt* (v3.12) [49]. Demultiplexed reads were filtered and trimmed at 275 bp for forward and 215 bp for reverse reads, and low-quality reads were discarded following protocols set at two expected errors (maxEE = 2), ambiguous nucleotides (maxN = 0), or erroneous bases (truncQ = 2) using the *DADA2* R-package (v3.22) [50]. Filtered and trimmed sequence reads were dereplicated to infer the amplicon sequences by removing sequencing errors*.* Denoised forwards and reverse reads were merged with a minimum overlap of 20 bp, yielding contigs of ~440–455 bp [51]. Recognizing that the PCR protocol could potentially generate chimeras [52, 53], we removed all potential chimeras at this point with *DADA2* [50]. From the 176 samples, we retrieved a total of 6175 unique contigs. Taxonomy was assigned using the adapted *nifH* ARB database v1.0.3 accessed in 2022 [54, 55], implementing a taxonomic naive Bayesian RDP classifier (v2.13) [56]. This database includes *nif*H homologs, which are nontarget amplicons coding for *ch1*L and *bch*X genes that were removed at this step, and we also excluded 780 ASVs with unassigned phylum. To minimize potential sequencing artifacts, the bacterial phyla *Bacteroidetes* and *Spirochaetes* were removed, represented by two and three ASVs, respectively. Eighteen ASVs identified as external contaminants were removed by performing ASVs frequency-based and prevalence-based methods with the *decontam* R-package (v3.21) [57]. The ASVs’ *nifH* gene sequences were aligned using the *DECIPHER* R-package (v3.12) [58], and a maximum likelihood phylogenetic tree was built under the GTR + GAMMA+I DNA evolution model with the *phangorn* R-package (v2.12.1) [59]. Additionally, we removed thirty-one samples with low sequencing depth (< 500 reads) and evaluated sequencing depth bias by correlating observed species richness and the read counts per sample (library size) using the Kendall method. Samples with low sequencing depth had significantly lower species richness (R^2^ = 0.746; *P* ≤ .001). We rarified to an even sequencing depth of 514 reads for downstream analysis, following normalization strategies for uneven library sizes [60]. The final dataset comprised 146 samples (Table S1), with a mean sequencing depth of 7003.9 reads (min = 571; max = 41 496) and a total of 4897 ASVs (3267 ASVs from 70 samples of *C. surinamensis*, and 2033 ASVs from 76 samples of *R. flaccida*).

### Host-specific diazotrophic microbiota

We used relative abundance and relative frequency of occurrence to identify the diazotrophic species predominantly associated with one epiphyllous bryophyte species compared to the other, with the indicator species analysis in the *labdsv* R-package (v2.1–0) [61]. We calculated an Indicator Value Index for each ASV between the two groups of samples: *Radula flaccida* and *Cololejeunea surinamensis*. Statistical significance was assessed using 1000 permutations, and ASVs with a maximum probability of *P* = .01 were considered as host-specific. These 75 ASVs identified were analyzed separately as the host-specific diazotrophs (Table S2). Additionally, Linear Discriminant Analysis Effect Size (LEfSe) [62] was used to identify diazotrophic taxa differentially abundant between host species and habitat size, employing the *microeco* R-package (v1.15) [63]. Differentially abundant taxa were identified using the test Kruskal-Wallis, with the effect size of significant phyla, genera, or ASVs determined using a threshold of LDA ≥ 2 (*P* > .05). We focused our diversity inferences and interpretations on the genera *Fischerella*, *Brasilonema*, and *Hassallia,* which were consistently identified by both methods. These taxa exhibited high specificity to the liverwort species, suggesting a strong host-association rather than mere differences in relative abundance.

### Analyses of diazotrophic community diversity

We estimated the relative abundance of diazotrophic ASVs based on the proportion of reads relative to the total sample sequence coverage [60, 64]. Alpha diversity was assessed using Hill numbers with three richness indices that accounted for differences in the weight of common taxa: (i) the Chao index, (ii) the Shannon exponential, which weights the ASVs by their frequency, and (iii) the Inverse Simpson’s diversity entropy, which considers ASV occurrence and relative abundances. Phylogenetic diversity was measured with the Faith index using the *picante* R-package (v1.8.2) [65, 66]. We inspected that the data was normally distributed with the Shapiro–Wilk test (*P* ≤ .001) and visual inspections using histograms and Q-Q plots. Homogeneity of variance was confirmed for all metrics using Levene’s test (*P* > .05). Generalized Linear Models (GLM) were used to evaluate the effect of the categorical variables of host species, habitat size, and habitat class (small vs large) on species richness indices, as a response variable.

Beta-diversity patterns of the diazotrophic community were explored using a Principal Coordinate analysis (PCoA). We assessed community composition using the non-phylogenetic Bray–Curtis dissimilarity index based on ASV relative abundance, and the weighted Unifrac distance, which incorporates ASVs’ phylogenetic relatedness, with the *vegan* (v2.7–1) [67] and *GUniFrac* (v1.8) [68] R-packages. The homogeneity of community dispersion was tested using permutation tests (PERMDISP2) with *vegan* [67]. We tested whether diazotrophic communities were more variable within the host in the same habitat size than across different habitat sizes. A permutation-based analysis of variance with 999 permutations was used to assess the significance of p values using the *car* R-package (v3.1–3) [69]. Diazotrophic community composition across host species, fragment size, and habitat classes was compared using permutational multivariate analysis of variance (PERMANOVA) with 999 permutations, implemented in *vegan* [67]. Pairwise comparisons were adjusted using Bonferroni correction.

### Metacommunity assembly processes

We implemented a null model-based approach to assess the assembly mechanisms shaping liverwort-associated diazotrophic metacommunities [70], using *microeco* [63]. The Raup-Crick dissimilarity metric based on Bray-Curtis distances (RCBray) was calculated from 1000 randomizations to quantify how much observed community composition deviates from what would be expected under random (stochastic) species distributions. We combined the RCBray with the β-nearest taxon index (βNTI) to infer the relative contributions of deterministic (e.g. selection) and stochastic (e.g. drift and dispersal) processes shaping microbial communities [66, 71]. Specifically, βNTI values are greater than +2 or lower than −2, indicating that deterministic processes, such as variable or homogeneous selection, dominate community assembly. In contrast, when βNTI < 2, observed phylogenetic turnover is as expected by chance, suggesting stochastic processes prevail, including ecological drift (−0.95 ≤ RCBray ≤0.95), dispersal limitation (RCBray >0.95), or homogenizing dispersal (RCBray < −0.95). We estimated the proportion of each assembly mechanism contributing to the community composition across 1-ha, 10-ha, and 100-ha fragments and continuous forests.

### Network analyses and detection of module hubs

Diazotrophic co-occurrence networks were analyzed using the *microeco* [63] and *igraph* (v2.1.4) R-packages [72]. Significant ASV associations (threshold *P* < .01) were estimated using Spearman correlations and the Bray–Curtis dissimilarity index. Networks were generated with the filter threshold set at ≥0.05% to detect ASV co-occurrences, balancing between the microbial community diversity and minimizing spurious correlations from extremely rare ASVs. Networks were constructed using 100 iterations, with renormalization and null distribution computations. Optimization of the correlation coefficient was performed using a random matrix theory-based method [73], which helps filter out low-abundance ASVs and reduces network complexity. Network metrics were compared to a null distribution using 1000 bootstrap iterations to assess statistical significance. Spearman correlation metrics were combined with the Brown method, and multiple-test corrections using the Benjamin Hochberg procedure, retaining edges with *q-values* < 0.05.

Four networks were constructed, corresponding to the two host species from small (1- and 10-ha; *C. surinamensis n* = 36, *R. flaccida n* = 37) and large (100-ha and continuous; *C. surinamensis n* = 34, *R. flaccida n* = 39) forest habitats. This approach enabled a comparison of network features between independent networks, providing insights into how co-occurrence patterns varied with a specific factor [38], here habitat size. Network properties: modularity, degree, betweenness, abundance, taxonomy, within-module connectivity (Zi), and among-module connectivity (Pi) were estimated using the *meconetcomp* R-package (v0.6.1) [74]. Network cohesion was used to quantify the degree of community connectivity across small and large habitats [75]. To detect module hubs or key species, ASVs (nodes) were classified according to their roles in the network by calculating their within-module connectivity (Zi) and among-module connectivity (Pi) [76]. Node roles were assigned based on their within- and among-module connectivity Zi-Pi plots, which separate the graphical space nodes into module hubs, network hubs, peripherals, and connectors [77].

We assessed network robustness by carrying out edge and node removal [78, 79], following the *meconetcomp* R-package [74]. Specifically, edges (edge_rand) and nodes (node_rand) were randomly removed. Edges were removed in decreasing order of weight (edge_strong), while nodes were removed in decreasing order of degree (node_degree_high). The final simulation corresponds to random key species (node_hub) removal. The average network efficiency (Eff) was measured as the total number of nodes and the shortest path between two nodes within the network following 10% removal steps, while the natural connectivity (Eigen) was measured as the average value that changes strictly monotonically with edge deletion [79].

## Results

### Alpha- and beta-diversity

We identified 4897 diazotrophic ASVs corresponding to four phyla, 11 classes, 17 orders, 28 families, and 40 bacterial genera (Table S3). The phylum Cyanobacteria comprised 73% of the ASVs; while Firmicutes made up 16%, Proteobacteria 10%, and Elusimicrobia <1%. The cyanobacterial Nostocales was the dominant order and relatively more abundant in *C. surinamensis*, followed by Clostridiales, which was more abundant in *R. flaccida* (Fig. S1). The most abundant genera were *Hassallia* (Tolypothrichaceae, Nostocales) in *C. surinamensis*, and *Fischerella* (Hapalosiphonaceae, Nostocales) in *R. flaccida*.

**Figure 2:**
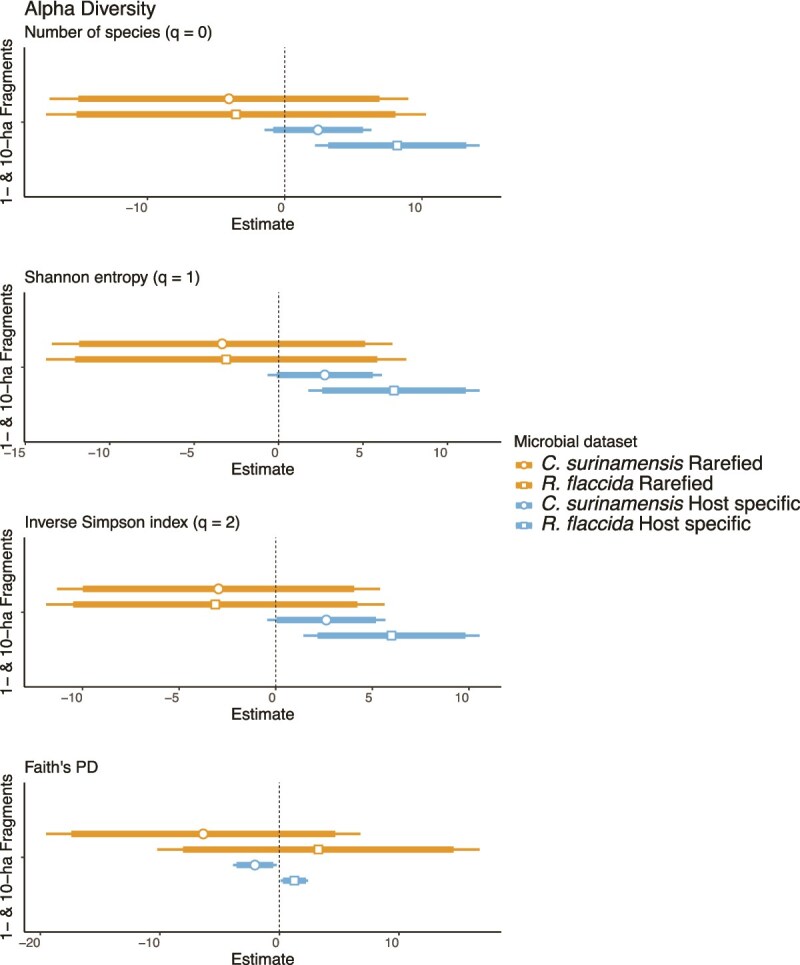
Alpha diversity of liverwort-associated diazotrophic communities across habitat sizes. Estimated effects of habitat size on the alpha diversity of diazotrophic communities associated with *Cololejeunea surinamensis* (*n* = 70) and *Radula flaccida* (*n* = 76) in an Amazonian fragmented landscape. Bars represent model-estimated coefficients (± standard error) from GLMs, indicating the direction and magnitude of richness changes between small and large habitat fragments. Two datasets are presented: Overall ASV diversity, a rarefied dataset considering ASVs with sequencing depth > 500 reads; and host-specific ASVs, depicting alpha diversity patterns specific to each liverwort species.

Higher richness was found in *C. surinamensis*, with observed species: $\bar{\textrm{X}} $ = 62.7 ± 4.16; Shannon: $\bar{\textrm{X}} $ = 49.9 ± 3.54; Simpson: $\bar{\textrm{X}} $ = 42.7 ± 3.28; compared to *R. flaccida* with observed species: $\bar{\textrm{X}} $ = 46.2 ± 5.29, Shannon: $\bar{\textrm{X}} $ = 36.9 ± 4.49 and Simpson: $\bar{\textrm{X}} $ = 31.69 ± 3.98 (Fig. S2; Tables S4 & S5). However, phylogenetic diversity did not differ between the two liverworts (Fig. S2). Microbial richness appeared slightly lower in small forest fragments (1- and 10-ha) compared to larger fragments (100-ha) and continuous forests, but the difference was not statistically significant (GLM, *P* = > .05; Fig. 2 (orange bars); Tables S4 & S5).

**Figure 3:**
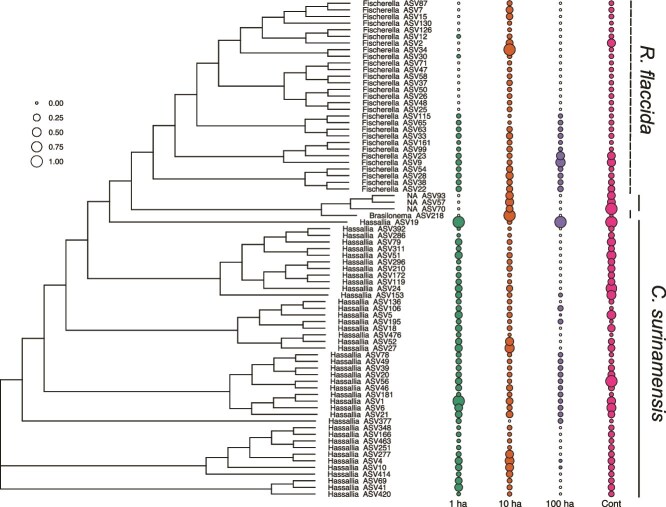
Maximum likelihood cladogram showing the phylogenetic relationships of liverwort host-specific associated microbiota to *C. surinamensis* and *R. flaccida*. Phylogenetic tree of identified ASVs through indicator species analyses, showing the relative abundance of each ASV across habitat size classes (1-ha, 10-ha, 100-ha forest fragments, and continuous forests). Circle sizes represent relative abundance, with open points indicating ASV absences in specific habitat classes.

Indicator species analyses identified 75 ASVs as host-specific. Fifteen of these ASVs were detected in more than 20% of host samples, indicating consistent occurrence across the dataset (Table S2). Forty-four ASVs in the genus *Hassallia* were associated with *C. surinamensis* (Fig. 3; Ind Value = 0.442–0.085, *P* ≤ .01; LEfSe: *P* ≤ .001; Fig. S3), with a single ASV, *Hassallia* ASV1, present in 40% of the samples. The genera *Nostoc* and *Brasilonema* were also significantly associated with *C. surinamensis* (LEfSe: *P* ≤ .001; Fig. S3). *Radula flaccida* was associated with 29 ASVs in the genus *Fischerella*, along with a single *Hassallia* ASV19 and *Brasilonema* ASV218 (Fig. 3; Ind Value = 0.145–0.092, *P* < .01; LEfSe: *P* < .01; Fig. S3). Although *Nostoc* ASVs were more abundant in *C. surinamensis* (Fig. S3), the genus was also present in *R. flaccida* in lower abundance, with two identified as key species based on the co-occurrence networks (Table 3). Host-specific diazotroph community exhibited similar richness between *C. surinamensis*: $\bar{\textrm{X}} $ = 10.52 ± 4.16, and *R. flaccida*: $\bar{\textrm{X}} $ = 9.27 ± 4.16 (Table S4). The GLM indicated that both host species had slightly higher richness of host-specific diazotrophic ASVs in smaller habitats than in continuous forests (t-value = 2.8, *P* < .001; Fig. 2 (blue bars); Fig. S2). Phylogenetic diversity was higher for *C. surinamensis*: $\bar{\textrm{X}} $ = 2.35 ± 0.5, compared to *R. flaccida*: $\bar{\textrm{X}} $ = 1.37 ± 0.25. Phylogenetic diversity in smaller habitats was lower for *C. surinamensis*, but higher for *R. flaccida* in 1-ha fragments (GLM: t-value = 2.17, *P* < .05). The relative abundance of the host-specific ASVs tended to decline in small habitats, while 16 ASVs for *R. flaccida* and three for *C. surinamensis* were absent in 1-ha fragments (Fig. 3). The absence of several of these host-specific ASVs in the single 100-ha fragment might be due to the limited number of this fragment size in the BDFFP landscape. Therefore, our comparisons focused on the broader contrast between small (1- and 10-ha) and the surrounding continuous forest habitats.

**Figure 4:**
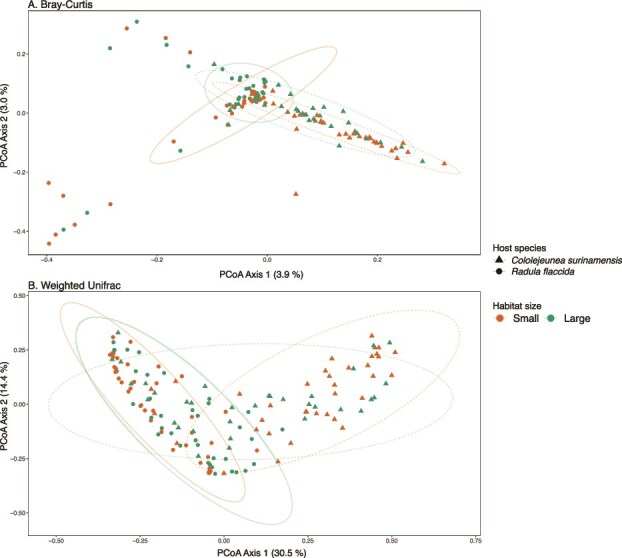
Variation in liverwort-associated diazotrophic community composition across habitat sizes and host species. PCoA ordination plots illustrating beta-diversity of diazotrophic communities associated with *C. surinamensis* (dashed line; *n* = 70) and *R. flaccida* (solid line; *n* = 76) across small forest fragments (1- and 10-ha) and large forests (100-ha fragments and continuous forests). (A) Non-phylogenetic Bray–Curtis dissimilarity and (B) phylogenetic Weighted Unifrac distances. Ellipses represent 95% confidence intervals. Both metrics were calculated using 4897 *nif*H ASVs.

Distinct diazotrophic communities were observed between the two bryophyte species in the ordination space PCoA (Fig. 4A, B, Table S6), PERMANOVA – Bray-Curtis: R^2^ = 0.02, *p* 0.001; Weighted UniFrac: R^2^ = 0.13, *p* 0.001. To a lesser extent, forest size explained between 2.5%–3% of the community variation, PERMANOVA—Bray–Curtis: R^2^ = 0.025, *P* = .001; Weighted UniFrac: R^2^ = 0.03, *P* = .01. Multivariate dispersion of the diazotrophic community was higher in *R. flaccida* than in *C. surinamensis* (Table S7). In 1-ha fragments, *R. flaccida* showed higher phylogenetic variation, but lower in 10-ha, compared to large habitats (Table S7).

The null model-based approach revealed that homogeneous dispersal was the dominant assembly mechanism shaping bryophyte-associated microbial communities across all habitat sizes (Table 1), particularly in smaller fragments (up to 63% in 1-ha). However, variable selection was higher in large habitats (27%–37%), indicating greater host selectivity in larger habitats compared to small habitats. Dispersal limitation and ecological drift were more pronounced in small fragments, but to a lesser extent compared to the proportion of homogenizing dispersal. In continuous forests, diazotrophic communities were shaped by both deterministic (selection) and stochastic (drift and dispersal) processes (Table 1).

**Table 1:** Relative contribution of community assembly processes based on Raup-Crick dissimilarity (RCBray) for liverwort-associated diazotrophic communities across habitat sizes. Values represent the percentage of each inferred ecological process (e.g. homogenizing dispersal, variable selection) across different forest fragment sizes and continuous forest sites. Sample size (*n*) refers to the total number of host–microbial communities from the two liverwort species, collected in 1-ha (*n* = 3), 10-ha (*n* = 3), 100-ha (*n* = 1), and continuous forest plots (*n* = 4).

**Processes**	1-ha	10-ha	100-ha	Continuous forest
Variable selection	11.26	21.92	37.18	27.12
Homogeneous selection	1.18	1.201	0	0.28
Dispersal limitation	12.27	7.96	2.56	5.71
Homogeneous dispersal	62.86	59.46	57.69	51.24
Drift	12.44	9.46	2.56	15.65
Sample size (*n*)	36	37	13	60

### Microbial network analyses

Co-occurrence networks had a similar number of nodes, but a higher number of edges in the larger habitats (Table 2. *C. surinamensis*: ∆ = 1321 and *R. flaccida*: ∆ = 1697 edges). Only 3% of the nodes were shared between networks of small and large habitats, with up to 20% unique to habitat size (Table 2). Most network edges were unique, with fewer shared between networks. For *R. flaccida*, the comparison of other network features was lower for small habitats (Table 2). Modularity was higher in small habitats for both species, with significantly higher positive cohesion in small habitats (*P* < .001).

**Table 2:** Liverwort associated diazotrophic microbe co-occurrence network topological attributes and between nodes and edges comparisons of the two host species in forests of different sizes small: fragments 1-ha, 10-ha, and large: 100-ha and continuous forests (sampling size is given for each network as the number of host-microbial communities).

	*C. surinamensis* Small (*n* = 36)	*C. surinamensis* Large (*n* = 34)	*R. flaccida* Small (*n* = 37)	*R. flaccida* Large (*n* = 39)
Vertex	607.00	644.00	569.00	551.00
Edge	5084.00	6405.00	3984.00	5681.00
Average degree	16.75	19.89	14.00	20.62
Average path length	3.76	4.01	1.04	3.72
Network diameter	8.00	10.00	3.00	9.00
Clustering coefficient	0.89	0.85	0.98	0.82
Density	0.03	0.03	0.02	0.04
Heterogeneity	0.38	0.36	0.52	0.35
Centralization	0.03	0.03	0.03	0.03
Modularity	0.90	0.87	0.95	0.85
Module hubs	12	5	0	2
Peripheral nodes	311	465	83	469
Unique number of nodes	495 (23.6%)	498 (23.8%)	444 (21.2%)	414 (19.8%)
Unique number of edges	5002 (24.3%)	6151 (29.9%)	3654 (17.8%)	5186 (25.2%)
Shared number of nodes	64 (3.1%)		62 (3%)	
Shared number of edges	37 (0.2%)		279 (1.4%)	

**Figure 5:**
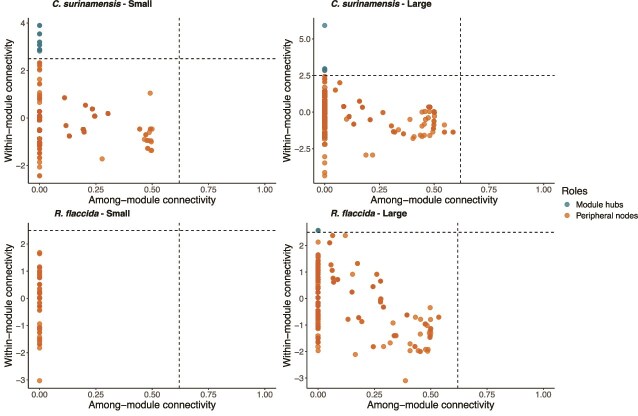
Network inference analysis Zi-Pi plots showing within-module (Zi) and among-module (Pi) connectivity of ASVs associated with *C. surinamensis* and *R. flaccida* in small forest fragments (1- and 10-ha) and large forests (100-ha fragments and continuous forests). Identified taxa roles for each ASVs are classified as module hubs (Zi > 2.5), and peripherals taxa (Zi < 2.5, Pi <0.65). Dashed lines delineate the boundaries of these functional roles. Key species identified based on the within- (Zi) and among-module connectivity (Pi) indicated as Module hubs.

Key species or Module hubs, such as taxa with high within-module connectivity, were identified based on network theory using Zi-Pi plots (Fig. 5). We observed in large habitats five key species for the host *C. surinamensis* and two for *R. flaccida*. While 12 key species were detected in small habitats associated with *C. surinamensis*, for *R. flaccida* no key species were found, with all diazotrophs classified as peripheral taxa (Fig. 5). The composition of key species, module association, as well as their relative abundance changed between networks of different habitat size (Fig. S4; Table 3). Specifically, *C. surinamensis* diazotrophs in small habitats had 11 *Hassallia* ASVs and one *Yangia* as key species, while in large habitats, one *Nostoc* ASV, and two ASVs of *Hassallia* and *Stigonema* each, were the key species for three different modules. These key species were present in higher relative abundance compared to peripheral taxa and were highly connected within their respective modules (Figs. 5 & S4). Two *Nostoc* ASVs were determined as key species restricted to large habitats for *R. flaccida* (Table 3).

**Table 3:** Key species (Module hubs) and taxonomic identity in three of the four networks of liverwort associated diazotrophic community for the host species *R. flaccida* and *C. surinamensis* in small and large habitats. Details are given for their corresponding network attributes and ASV relative abundance. No key species were found in the network of *R. flaccida* small habitats.

Network	Taxa	Degree	Betweenness centrality	Closeness centrality	Eigenvector centrality	Abundance	Module	Z-value
*R. flaccida* Large habitats	*Nostoc* ASV77	30	2864.281	0.000543	7.52E-06	0.189	M4	2.572
	*Nostoc* ASV112	30	2864.281	0.000543	7.52E-06	0.085	M4	2.572
*C. surinamensis* Small habitats	*Hassallia* ASV147	26	1.52E+03	0.000663	9.12E-17	0.097	M3	3.198
	*Hassallia* ASV202	34	2.92E+03	0.000771	1.12E-16	0.162	M5	3.899
	*Hassallia* ASV228	26	1.52E+03	0.000663	7.75E-17	0.070	M3	3.198
	*Hassallia* ASV256	34	2.92E+03	0.000771	7.95E-17	0.146	M5	3.899
	*Hassallia* ASV310	32	1.77E+03	0.000925	0.00E+00	0.162	M6	3.543
	*Yangia* ASV330	27	5.34E+03	0.000673	2.95E-16	0.065	M4	2.821
	*Hassallia* ASV406	32	1.77E+03	0.000925	0.00E+00	0.162	M6	3.543
	*Hassallia* ASV468	28	9.00E+01	0.000525	3.22E-16	0.200	M4	3.076
	*Hassallia* ASV516	25	1.40E+03	0.000733	9.65E-17	0.070	M3	2.878
	*Hassallia* ASV562	28	9.00E+01	0.000525	3.51E-16	0.162	M4	3.076
	*Hassallia* ASV603	25	1.40E+03	0.000733	9.16E-17	0.081	M3	2.878
	*Hassallia* ASV711	27	2.02E+03	0.000717	1.72E-16	0.054	M7	2.873
*C surinamensis* Large habitats	*Hassallia* ASV106	29	5695.336	0.000499	1.20E-02	0.132	M6	2.846
	*Hassallia* ASV195	29	5695.336	0.000499	1.20E-02	0.052	M6	2.846
	*Stigonema* ASV322	34	3315	0.000335	4.11E-04	0.350	M9	2.963
	*Stigonema* ASV451	34	3315	0.000335	4.11E-04	0.315	M9	2.963
	*Nostoc* ASV996	37	342	0.0388	0.00E+00	0.292	M7	5.920

**Figure 6:**
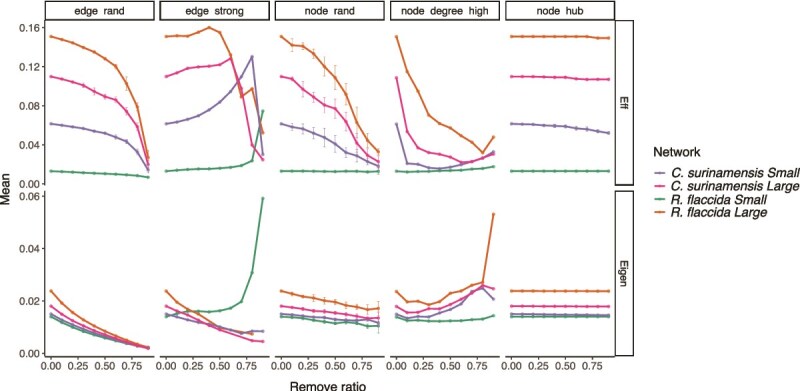
Network robustness of liverwort-associated diazotrophic communities across habitat sizes. Network robustness analysis based on average network efficiency (Eff) and natural connectivity (Eigen) under simulated node and edge removals in diazotrophic communities associated with *C. surinamensis* and *R. flaccida* in small forest fragments (1- and 10-ha) and large forests (100-ha fragments and continuous forests). Simulations include random edge removal (edge_rand), random node removal (node_rand), edge removal in decreasing order of weight (edge_strong), node removal in decreasing order of degree (node_degree_high), and random key species (node_hub) removal. Separate networks are represented by color-coded lines in each vertical panel.

The random removal of nodes and edges decreases network connectivity and robustness, regardless of liverwort species or habitat size (Figs. 6, S5). When edges were removed by decreasing weight, robustness decreased tandemly for large habitats, while it initially increased in small habitats before collapsing after 80% of edges were removed. The removal of nodes with a high degree of interaction had similar effects, with a substantial network robustness decrease at the 80% interval. The removal of node hubs didn’t affect the robustness of the network. Overall, networks in large habitats exhibited higher robustness than those in small habitats (Fig. 6).

## Discussion

### Homogenizing dispersal of liverwort-diazotroph metacommunities

Liverwort-associated diazotroph exhibited distinct host-specific compositions. *Cololejeunea surinamensis* was primarily associated with the genus *Hassallia*, while *R. flaccida* was associated with *Fischerella.* We investigated the dynamics of the diazotrophic community and network at both the meso-scale (within forest sites <0.01 km^2^) and macro-scale (across 1000 km^2^) in the experimentally fragmented forest landscape of the BDFFP. Contrary to expectations under dispersal limitation, diazotrophic richness associated with both liverwort species did not consistently decline in smaller forest fragments. This pattern was consistent with species assembly processes of strong microbial mixing (homogenizing dispersal). However, dispersal limitation and ecological drift were more pronounced in small fragments, reflecting increased stochasticity compared to continuous forests. Notably, both dispersal limitation and homogeneous dispersal co-occurred in small fragments, suggesting that while efficient local dispersal occurred, broader landscape connectivity remained restricted. The assembly mechanisms shaping host-microbe associations in plants are complex and may be highly dependent on habitat size [19, 20, 31]. The habitat-dependent nature of assembly mechanisms suggests that shifts in habitat environmental conditions and connectivity, such as those driven by habitat fragmentation, may significantly alter plant-associated microbial communities [24, 25, 29].

The surrounding forest matrix of the BDFFP has changed to a 20 to 35-year-old secondary forests, diminishing the effects of fragmentation for small fragments [43] and promoting species recovery [28, 45]. A similar rapid natural recovery in the diversity of soil diazotroph communities was observed, comparable to that of primary forests, after pasture transitioned to secondary forests [15, 16]. The surrounding continuous forests may facilitate a biodiversity spillover into smaller fragments and secondary forests. This could help explain the similar richness levels and the high proportion of homogenizing dispersal observed across the landscape [19, 31, 32, 80]. In continuous forests, the diazotrophic community appeared to be less constrained, with both liverwort species showing slightly higher diazotrophic richness and resulting in more homogeneous communities. Continuous forests exhibited a more balanced interplay of deterministic and stochastic processes, and the higher proportion of variable selection suggested stronger host filtering effects [20].

### Microbial network stability and key species

Network analyses showed that habitat size has an impact on the stability and patterns of liverwort diazotrophs co-occurrence. In large and continuous habitats, networks showed higher connectivity and robustness. Diazotrophic communities are likely more stable due to the positive feedback of the among-module hubs connectivity with multiple interactions with peripheral nodes (generalist taxa), buffering against random taxa loss [38, 81]. In contrast, the greater modularity in small fragments indicates a breakdown of cohesive interactions among modules and network connectivity.

We identified key species that had a pivotal role in maintaining the structure and function of host diazotrophic networks. Key species often act as ecological buffers, maintaining network stability in the face of environmental disturbance and processes [41]. In the network of *C. surinamensis* from continuous forests, key species of the same genus formed distinct modules, with a single *Nostoc* ASV exhibiting high within-module connectivity. Whereas in fragmented forests, the species exhibited a reorganization into different modules, suggesting a breakdown of stable genus-level co-occurrence associated with reduced host specificity. The *R. flaccida* microbiota was even more susceptible to habitat size, where a co-occurrence network of small fragments had high modularity, and key species were absent. The lower robustness in small fragments suggests that even minor disruptions leading to a random loss of nodes and edges, or specific removal based on the taxon role and connectivity, could lead to the collapse of entire networks [77].

Our results support the notion that the reduced size of forest fragments disrupts microbial co-occurrence networks, leading to an imbalance in microbial associations and the loss of key species that could further impair metabolic functions. Habitat fragmentation can exacerbate the loss of key species and their functions, especially along the edges with more open, drier conditions [16, 45, 82], which have been related to reduced bryophyte abundance and altered microbial communities [83]. Seasonal shifts and host-specific variations in diazotrophic communities have been linked to fluctuating N-fixation rates as well [6]. These shifts, potentially associated with abiotic factors, complicate predicting how liverwort-diazotroph associations will respond to land-use change. In addition, the impact on the function of key N-fixing species, which are critical for nutrient cycling in tropical forests [15, 16, 22], remains to be explored [8].

### Liverwort-diazotrophic association

Liverworts in tropical forests may play a much greater role than previously recognized in maintaining forest ecosystem functions, particularly through their association with nitrogen-fixing bacteria [5, 8, 11, 12, 14, 84, 85]. In Costa Rican forests, epiphyllous bryophytes enhance BNF rates and act as a long-term (up to 180 days) nitrogen reservoir in the understory [11, 12]. Our findings suggest community-wide changes in liverwort-diazotroph associations in small forest fragments, which may compromise their contribution to BNF. Quantifying the functional contributions of epiphyllous bryophytes to phyllosphere biogeochemical cycles requires integrated approaches. Combining metagenomics with in-situ nitrogen fixation assays (e.g. acetylene reduction and stable isotope techniques) could effectively elucidate how microbial diversity and interactions drive ecosystem functions [6, 12].

Although fragmentation effects on macro-communities are well documented, understanding the responses of microbial functional groups like diazotrophs and their key role in BNF is vital for assessing forest ecosystem resilience. Our study focused on two liverwort species and associated diazotrophs, yet bryophytes harbor a distinct and complex associate microbial community [8]. For example, differences in diazotrophic community composition associated with boreal forest moss species were related to their differential nitrogen fixation rates [7, 9]. Bryophyte-associated diazotrophs contribute up to 35% to the nitrogen input in boreal environments [84] and tropical forests [6, 11, 12]. Despite their ecological importance, the consequences of shifts in key species and network stability on their potential ecosystem functions, such as N-fixation, are an emerging area of research with implications for forest dynamics and conservation [5, 6, 21, 22]. While our network analyses identified key species, their functional contributions to N-fixation remain speculative as the abundance of diazotrophic taxa is not an accurate predictor of nitrogen fixation [86, 87]. Experimental approaches using synthetic communities (SynComs) or synthetic ecosystems (SynEcos), offer a promising avenue to disentangle the roles of microbial species and plant-microbe association in maintaining functional resilience [88].

Our findings highlight the complexity that habitat size and connectivity play in the host microbe metacommunity dynamics in tropical forest ecosystems. The results suggest that larger, continuous habitats support more stable microbial networks, with key species of microbes potentially playing a crucial role in maintaining plant-diazotroph associations. In contrast, in small forest fragments, the proportion of assembly mechanisms such as dispersal limitation and drift, identified using Raup-Crick null models, may contribute to changes in liverwort-associated diazotrophic community. The simulated loss of microbial taxa resulted in plant-diazotroph associations becoming more vulnerable to network collapse, with lower robustness in small forest fragments. In conclusion, our findings highlight the urgent need for targeted forest conservation and management strategies that prioritize not only the preservation of macro-species diversity but also the maintenance of key plant-microbe associations, which are essential for sustaining ecosystem functions and resilience.

## Supplementary Material

03_Sierra_etal_SupplementaryMaterial_ycaf173

04_Supplementary_tables_nov24_ycaf173

## Data Availability

Genetic data: Raw sequence data were deposited in the NCBI Sequence Read Archive (SRA) with their respective accession numbers under the BioProject: PRJNA1169898. The metadata, microbial community matrix, and the scripts used for the analyses that support the findings of this study are openly available at: https://github.com/adrielmsierra/LiverwortAssociatedDiazotrophs.
