# Supplementary material for: Assembly and co-occurrence networks of nitrogen-fixing bacteria associated with epiphyllous liverworts in fragmented tropical forests
Source: ISME Commun. 2025 Sep 27;5(1):ycaf173. doi: 10.1093/ismeco/ycaf173 (PMC12560778; doi:10.1093/ismeco/ycaf173)
Supplement: 03_Sierra_etal_SupplementaryMaterial_ycaf173 [file 03_sierra_etal_supplementarymaterial_ycaf173.pdf]

Supplemental material

Assembly and co-occurrence networks of nitrogen-fixing bacteria associated with epiphyllous liverworts in fragmented tropical forests

Running title: Assembly of Liverwort N-Fixing Bacteria

Adriel M. Sierra[1,2,3*], Dennis Alejandro Escolástico-Ortiz [1,2], Charles E. Zartman[3], Nicolas Derome[1,2], Connie Lovejoy[1,2] & Juan Carlos Villarreal A.[1,2*]

[1] Département de Biologie, Université Laval, G1V 0A6, Québec (QC), G1V 0A6, Canada

[2] Institut de Biologie Intégrative et dês Systèmes (IBIS), Université Laval, Québec (QC), G1V 0A6, Canada

[3] Instituto Nacional de Pesquisas da Amazônia, Departamento de Biodiversidade, Av. André Araújo, 2936, Aleixo, CEP 69060-001, Manaus, AM, Brasil

AMS: amsip1@ulaval.ca, (orcid: https://orcid.org/0000-0001-9900-1350)
DOE: escolasticodennis@gmail.com, (https://orcid.org/0000-0002-3001-6148)
CEZ: chaszartman@gmail.com, (orcid: https://orcid.org/0000-0001-8481-9782)
ND: nicolas.derome@bio.ulaval.ca, (https://orcid.org/0000-0002-2509-6104)
CL: connie.lovejoy@bio.ulaval.ca, (https://orcid.org/0000-0001-8027-2281)
JCVA: jcvil9@ulaval.ca, (orcid: https://orcid.org/0000-0002-0770-1446)

*Corresponding authors
Adriel M. Sierra
Juan Carlos Villarreal A.
Département de Biologie, Pavillon C.E. Marchand 2212,
Université Laval,
Québec (Québec), G1V 0A6,
Canada

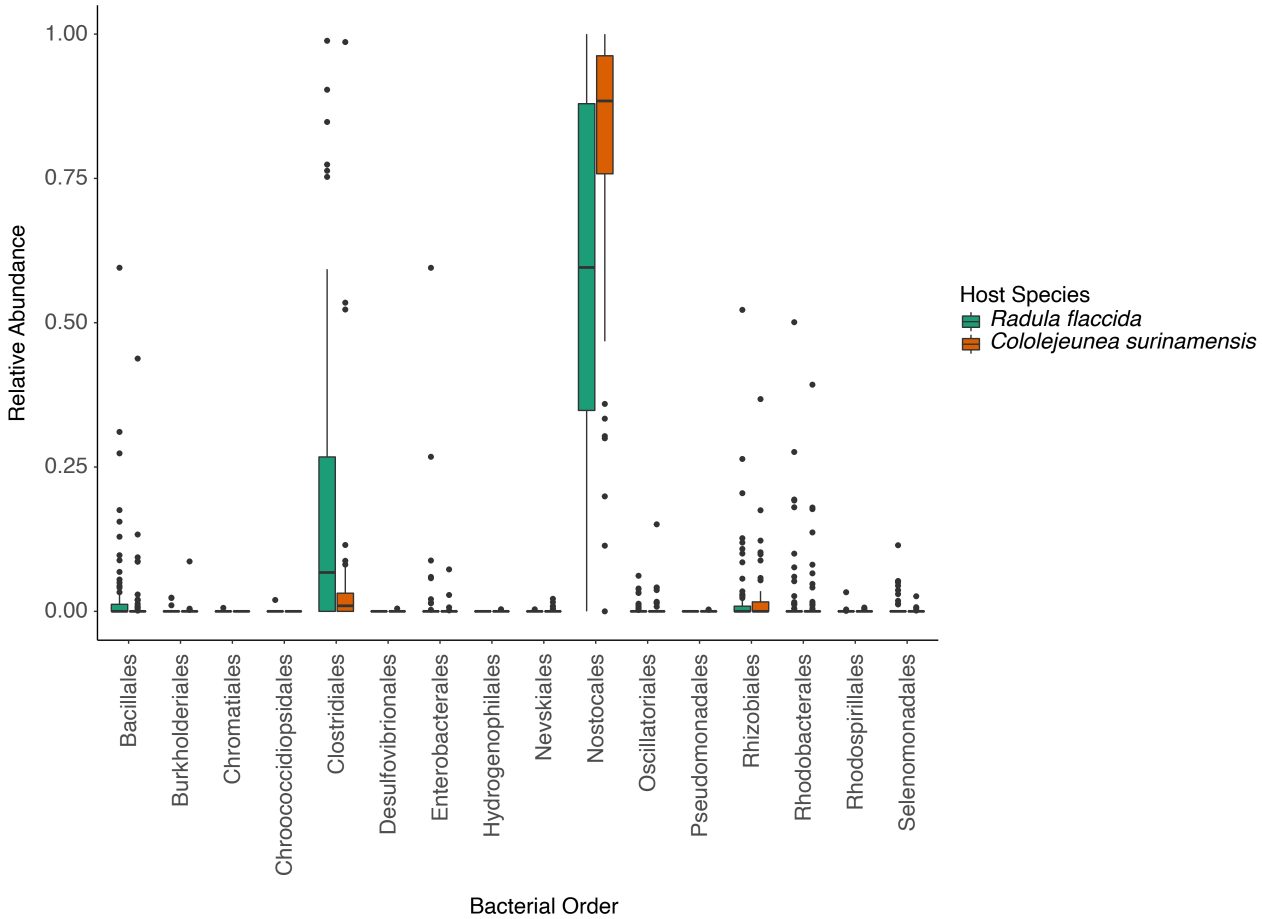

Figure S1. Relative abundance of diazotrophic bacterial orders in liverwort hosts. Boxplots showing the relative abundance of diazotrophic ASVs classified at the order level, separated by liverwort host species. *R. flaccida* (green, left) and *C. surinamensis* (orange, right) display distinct bacterial order compositions, highlighting host-specific patterns in microbial community

structure.

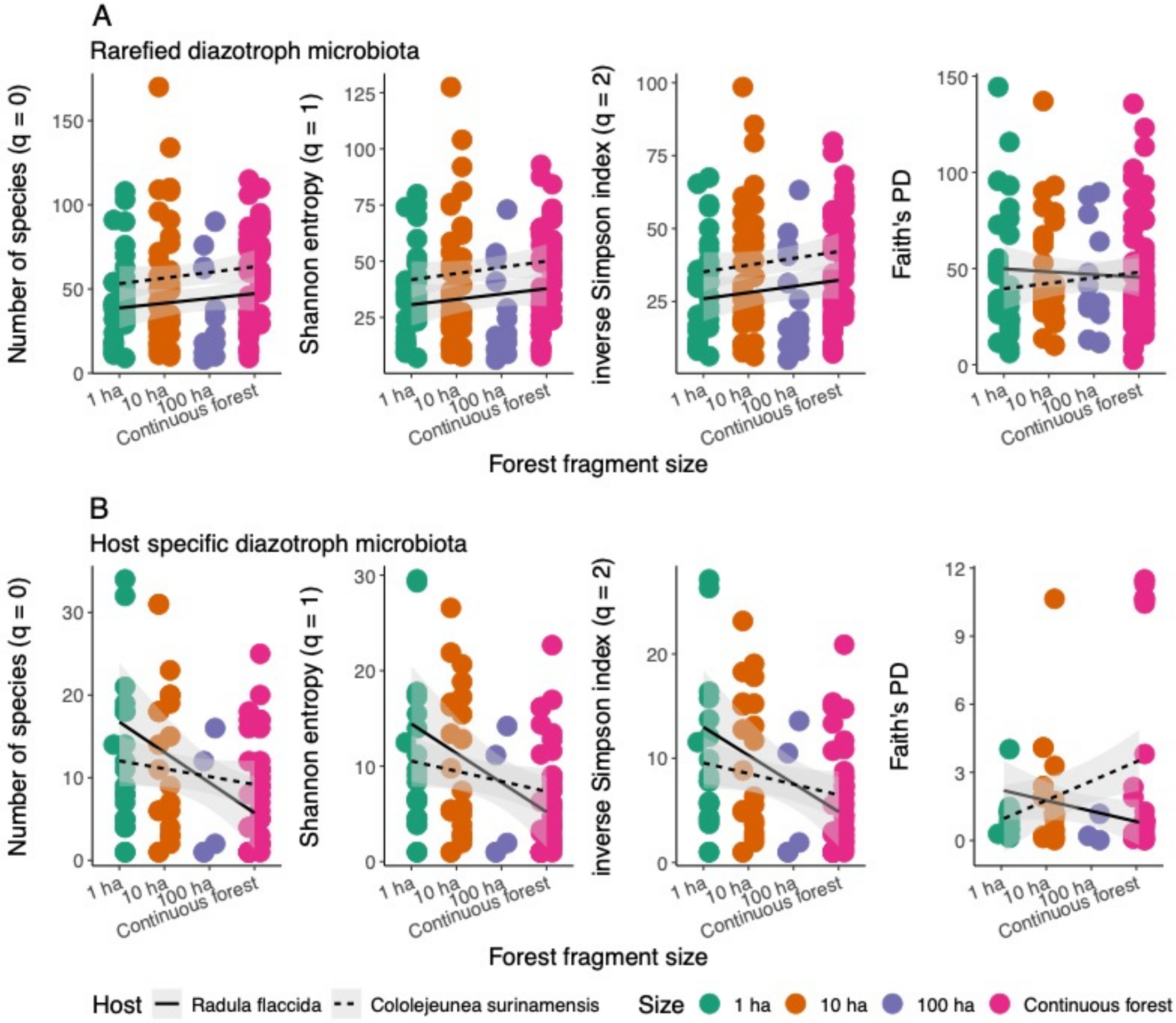

Figure S2. **Alpha diversity of the liverwort diazotrophic community in relation to habitat size.** (A) Alpha diversity metrics for the rarefied dataset and (B) for host-specific taxa identified through indicator species analysis. Each vertical panel (from left to right) presents the observed species richness, Shannon entropy index, inverse Simpson index, and Faith's phylogenetic diversity index. Linear regression lines illustrate the relationship between habitat size and alpha diversity for the two liverwort hosts, *R. flaccida* and *C. surinamensis*.

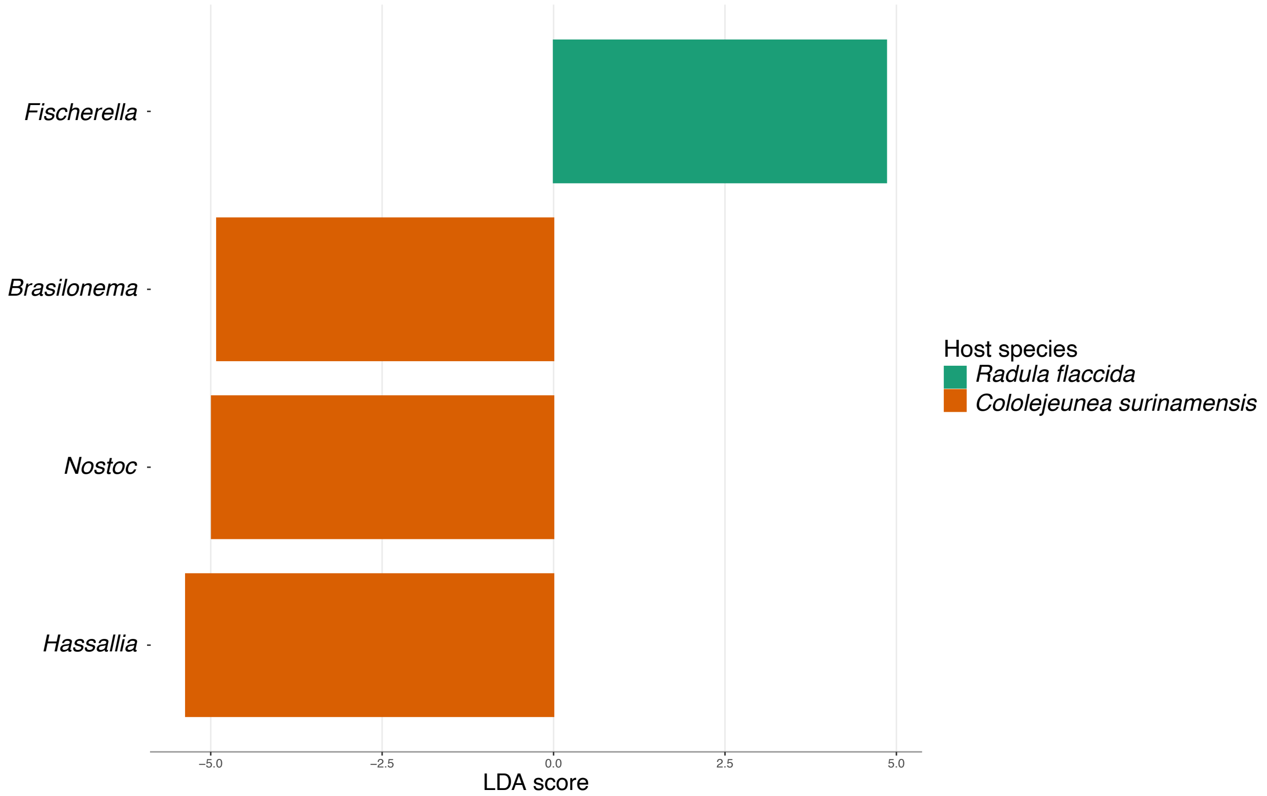

Figure S3. Differential abundance of diazotrophic genera between the liverwort host species *Cololejeunea surinamensis* and *Radula flaccida*. The analysis was performed using Linear Discriminant Analysis Effect Size (LEfSe), with a significance threshold of Linear Discriminant Analysis (LDA) score ≥ 4.

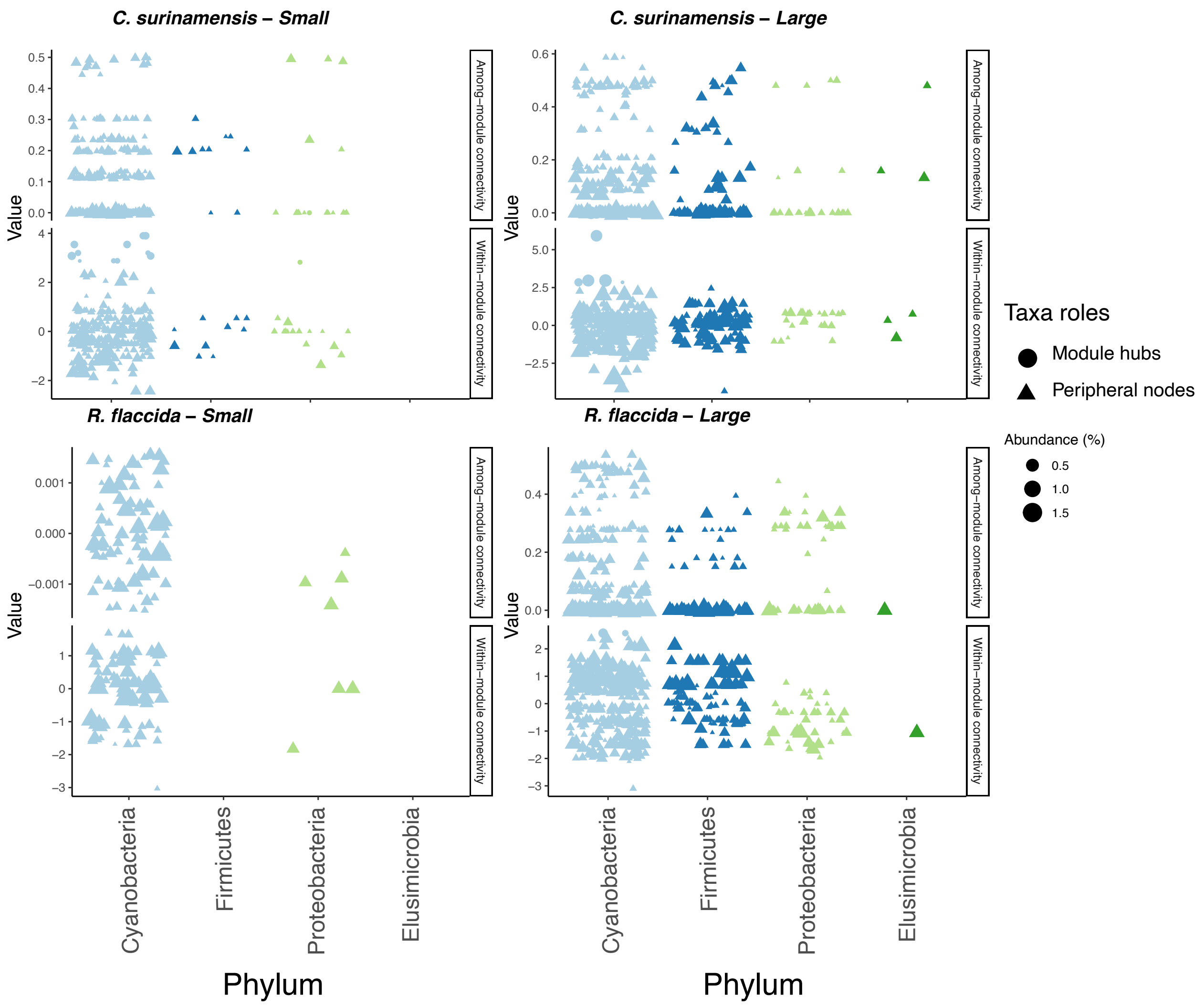

Figure S4. Microbial co-occurrence network features and taxa roles of the diazotrophic community associated with the liverwort host species *Cololejeunea surinamensis* and *Radula flaccida* in small (1- and 10-ha) and large (100-ha fragment and continuous forests) forest fragments. ASV profiles are classified by phylum in the four networks, with the y-axis representing their specific within- and among-module connectivity values. The figure is presented in two horizontal panels for each network. Point shapes represent taxa roles (module hubs and peripheral nodes), while point sizes indicate the relative abundance of the ASVs in the community.

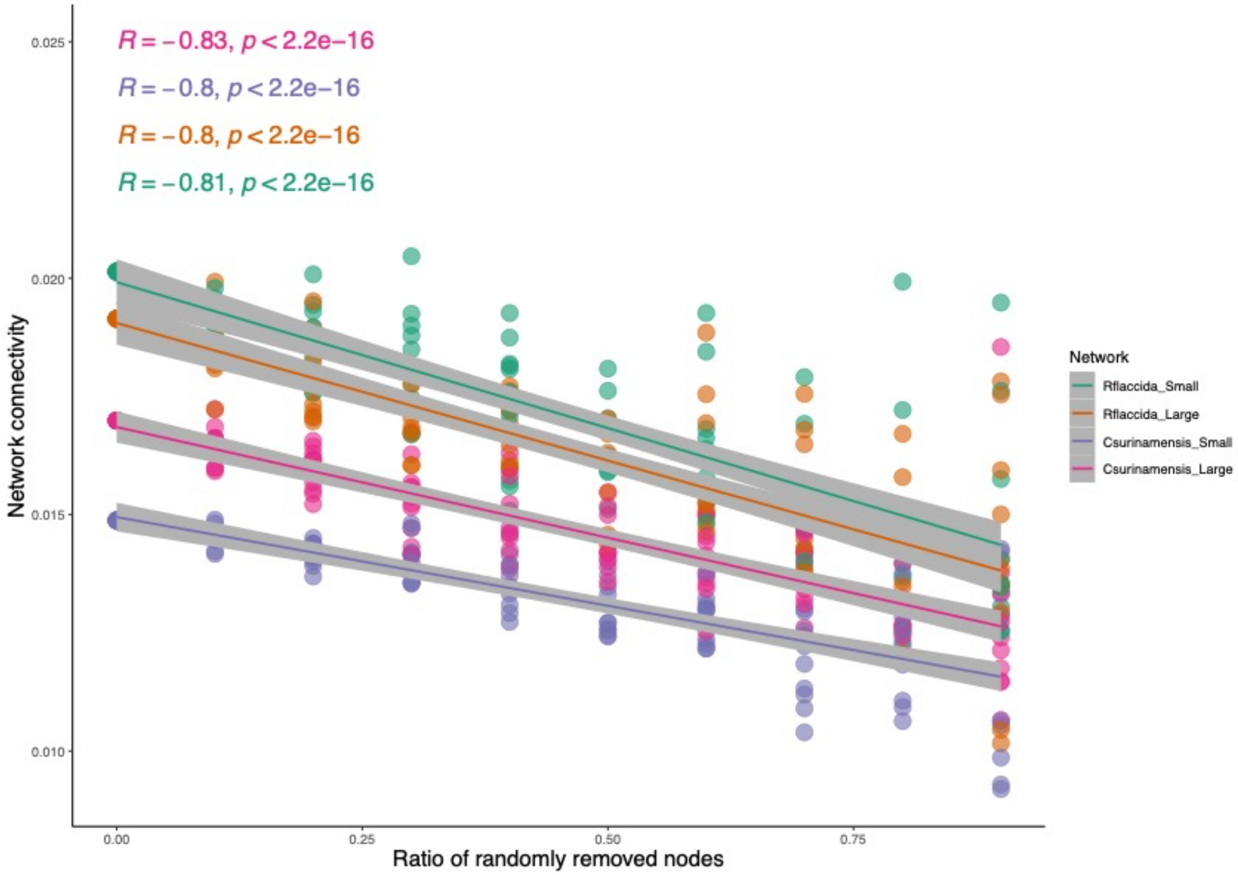

Figure S5. Linear regression between microbial co-occurrence network connectivity and the ratio of randomly removed nodes for the liverwort diazotrophic community associated with the host species *Cololejeunea surinamensis* and *Radula flaccida* in small (1- and 10-ha) and large (100-ha fragment and continuous forests) forest fragments. The figure includes the linear regression equation, R, and p-values for each regression.
